# Factors related to employment in childhood cancer survivors in Japan: A preliminary study

**DOI:** 10.3389/fped.2022.961935

**Published:** 2022-12-05

**Authors:** Kyoko Kobayashi, Yasushi Ishida, Michiyo Gunji, Kyoko Nagase, Yuri Yoshimoto-Suzuki, Yosuke Hosoya, Daisuke Hasegawa, Atsushi Manabe, Sachiko Ohde, Miwa Ozawa

**Affiliations:** ^1^ Department of Child & Family Health Nursing, St. Luke's International University, Tokyo, Japan; ^2^ Department of Pediatrics, Ehime Prefectural Hospital, Ehime, Japan; ^3^ Department of Nursing, St. Luke's International Hospital, Tokyo, Japan; ^4^ Department of Pediatrics, St. Luke's International Hospital, Tokyo, Japan; ^5^ Course of Advanced and Specialized Medicine, Graduate School of Medicine, Juntendo University, Tokyo, Japan; ^6^ Department of Pediatrics, Hokkaido University, Hokkaido, Japan; ^7^ Division of Epidemiology, Graduate School of Public Health, St. Luke's International University, Tokyo, Japan

**Keywords:** childhood cancer, survivors, employment, self-management, late-effect, transition readiness, academic achievement

## Abstract

**Purpose:**

Previous research has revealed vocational and academic difficulties in childhood cancer survivors, and explored impact of survivors' medical history and physical function on vocational and academic status. However, we often encounter survivors with similar diagnoses and late effects but different academic or employment statuses. This raises the question of what affects academic attainment and employment other than treatment or late effects. This study aimed to explore factors associated with childhood cancer survivors' employment status and academic achievement.

**Methods:**

Comprehensive health check-up and questionnaire survey were conducted for 69 survivors who were over the age of 18 and participated in St. Luke's Lifetime cohort study. We obtained survivors' biological function using comprehensive health check-up, neurocognitive states, quality of life, transition readiness, and family function. We conducted univariate analysis (Mann–Whitney *U* tests or chi-square tests) to compare the differences between the regular workers/students and non-regular workers/unemployed groups. The variables with *p*-values <0.1 were used as independent variables multivariate logistic regression to explore predictors of employment status and academic attainment.

**Results:**

Result of the univariate analysis, intelligence quotient, SF-8 PCS, transition readiness, family function were used for multivariate logistic regression as independent variables. The stepwise likelihood method was conducted; intelligence quotient (odds ratio [OR] = 1.100; 95% confidence interval [CI] 1.015–1.193; *p* = 0.021), transition readiness (OR = 0.612; 95% CI 0.396–0.974; *p* = 0.038), and family function (OR = 2.337; 95% CI 1.175–4.645; *p* = 0.015) were found to be associated with survivors' regular workers/students in the final regression model.

**Conclusion:**

Long-term follow-up of pediatric cancer survivors requires the provision of total care, which supports physical, psychological, and social functions to improve health, readiness for transition to self-management, and family functioning.

## Introduction

The survival rate for childhood cancer has improved, and the survival rates in Japan and Western countries have exceeded 80%. In Japan, the number of survivors of childhood cancer is uncertain, however an estimated 150,000—approximately one in 180 young adults at twenties—is regarded as childhood cancer survivors (CCSs). At the same time, various physical late effects, such as gonadal dysfunction, hypertension, or cognitive dysfunction caused by cancer and its treatment, have been reported (1, 2). As the measures and support for such challenges, there are 15 cancer centers which provide long-term follow-up care based on the guidelines set forth by the Japan Children's Cancer Group.

On the other hand, support for the developmental challenges such as academic achievement and/or low rate of employment which occurred in combination with late effects, long school absences due to treatment, and family factors is under-developed. Previous studies, including a population-based cohort study, have reported that academic attainment is lower in CCSs than in healthy peers; chemotherapy has been identified as a proximate cause of poor educational attainment according to some studies (3–6). The educational background of CCSs with multiple physical conditions is lower than those without those conditions (7). There is evidence that academic achievement is lower in younger-onset cases (8, 9). However, other reports have shown that age of onset is not relevant (10).

In terms of employment, some population-based studies have reported that CCSs are less likely to be working compared with peers (11, 12). French researchers have reported that the rate of CCSs seeking jobs was lower than that of the general French population, and the rate of CCSs having unstable employment was significantly higher (13). CCSs were unemployed for health reasons, and there were no significant differences between CCSs and the general population in terms of unemployment unrelated to health or those in work (14). In central nervous system tumor CCSs who underwent radiation therapy, the lower the age at diagnosis, the higher the number of late effects reported as risk factor for unemployment (7, 11–14). However, there is no existing consensus of risk factors of psychosocial function on academic attainment or employment metrics of Japanese CCSs. Furthermore, since there are large differences in income, social security, and employment stability between regular and non-regular employment in Japan, it is necessary to consider the type of employment when examining predictors of employment.

Empirically, CCSs with similar diagnoses and late effects yet different academic/employment statuses are often encountered. This raises the question of what affects academic attainment and employment other than treatment or late effects. A combination of biopsychosocial functions could influence childhood cancer survivors' academic attainment and employment; therefore, it is necessary to consider survivors' biological and psychosocial functions as influencing factors.

As in the Erice Statement (15, 16), the long-term goal of the cure and care of a child with cancer is that he/she becomes a resilient and autonomous adult with optimal health-related quality of life, accepted in society at the same level as his/her age peers, and to provide systematic support to empower CCSs and their families' adjustment and coping strategies to overcome future challenges in all aspects of life: in education, in work, and in family life. Therefore, the study aimed at ascertaining the factors associated with employment state and academic achievement among CCSs in Japan.

## Materials and methods

### Participants

The participants were 68 CCSs who correspond to all the subjects who participated in the St. Luke's Lifetime Cohort Study. Participants of the St. Luke's Lifetime Cohort Study were those over the age of 18 at the time of the participant, who had completed at least 5 years post treatment, and who knew their diagnosis.

We had calculated sample size using G* power for Mann-Whitney test (effect size = 0.5, *α* error = 0.05, power 0.95) and logistic regression (*α* error = 0.05, power 0.80), and it were 92 and 55, respectively. Therefore, it is possible to erroneously determine that there is no difference between the averages of data that are truly different as current study is a preliminary study with small sample.

#### The St. Luke's Lifetime Cohort Study

The St. Luke's Lifetime Cohort Study is a study of CCSs who were diagnosed with childhood cancer and treated at St. Luke's International Hospital, or a hospital affiliated with St. Luke's International Hospital. Inclusion criteria were, in addition to the above, 5 years after completion of treatment, 18 years of age or older, and able to complete a self-administered questionnaire. The St. Luke's Lifetime Cohort Study consists of a comprehensive health check-up with general and survivor-specific health examinations, psychological check-up, and tests to evaluate the state of late-effects and a questionnaire survey, which is conducted every 5 years.

CCSs meeting the inclusion criteria were briefed on the cohort study by a physician. If the survivor agrees to participate, provide written consent. After that, a questionnaire and schedule for a comprehensive health check-up were mailed to the CCSs.

On the comprehensive health check-up day, CCSs answered questionnaire provided prior to the check-up day and underwent a comprehensive medical examination at St. Luke's International Hospital Affiliated Clinic, Center for Preventive Medicine. For psychological check-up, CCSs revisited the hospital for intelligence quotient (IQ) tests on a day different from their health check-up. After the health check-up and IQ tests, all participating survivors received their IQ test results and feedback forms containing recommendations for a healthy lifestyle and long-term follow-up outpatient visits.

### Measurements

All data were obtained from the St. Luke's Lifetime Cohort Study including academic attainment, employment status, medical history, physical condition, and psychosocial condition.

#### Dependent variable: state of academic attainment or working status

We divided the CCSs in two sub-groups. One consisted of students (*n* = 22) and regular workers (*n* = 37), another consisted of non-regular workers and unemployed (*n* = 9).

In the questionnaire survey of the St. Luke's Lifetime Cohort Study, the participants stated whether they were currently working, pursuing a degree (graduate school, college, vocational school, or junior college). Those who were working were asked employment type (regular or non-regular), and those enrolled in school asked institution type (graduate school, college, vocational school, or junior college). Classification of schools and employment was based on the census classification of the Ministry of Internal Affairs and Communications (17).

Since financial and social participation are important indicators from the perspective of children's independency. And there are large differences in income, social security, and employment stability between regular and non-regular employment in Japan, it is necessary to consider the type of employment when examining predictors of employment. In adittion to that, academic achievement higher than high school significantly affect getting regular job (18). And attending school as a fulltime student is considered as regular social participatory. Therefore, we combined regular workers and students as one group and non-regular worker was another.

#### Physical indicators

##### Medical history

We collected medical history data from long-term follow-up summary which was written by the attending physician and was provided by the CCSs. In this study, we include CCSs' diagnosis and treatment (chemotherapy, surgery, radiation, or hematopoietic cell plantation).

##### Physical condition

Health condition of survivors were obtained from the comprehensive medical examination of St. Luke's Lifetime Cohort Study, which included tests for lifestyle-related diseases, blood and urine sample tests, an electrocardiogram, physiological tests (hearing test, chest x-ray test), diagnostic imaging (abdominal ultrasonography), and barium-based stomach examination. Additionally, provisions were made for women who wished to undergo gynecological examination, such as cervical screening and pelvic examination.

CCSs' physical conditions were classified based on the National Cancer Institute's Common Terminology Criteria for Adverse Events (the CTCAE) version 4 (19, 20). The CTCAE is a descriptive terminology which can be utilized for Adverse Event reporting (19, 20). A grading (severity) scale is provided for each Adverse Event term. The CTCAE displays Grades 1 through 5 with unique clinical descriptions of severity for each Adverse Event based on this general guideline: Grade 1 Mild; asymptomatic or mild symptoms; clinical or diagnostic observations only; intervention not indicated. Grade 2 Moderate; minimal, local or noninvasive intervention indicated; limiting age-appropriate instrumental Activities of Daily Living (ADL). Grade 3 Severe or medically significant but not immediately life-threatening; hospitalization or prolongation of hospitalization indicated; disabling; limiting self-care ADL. Grade 4 Life-threatening consequences; urgent intervention indicated. Grade 5 Death related to Adverse Event (19, 20). We developed modified version of the CTCAE to better accommodate the grading of Japanese childhood cancer survivors (21). For this study, we considered survivors who have grades 1 or severe defined were those who have condition.

#### Psychosocial indicators

In terms of psychosocial indicators, we collected information regarding IQ, quality of life (QoL), depression as a psychological indicator, and family function as a social indicator.

##### IQ

The IQ of the participants was evaluated by psychologists using Wechsler Adult Intelligence Scale-IV as an index of neuro-cognitive function. We used the IQ score as continuous variable.

##### Qol

We used the Japanese version of the Short Form-8 (SF-8) questionnaire (22), a comprehensive health-related QoL scale, which consists of eight items: physical function, daily role function, body pain, overall health, vitality, Social life function, daily role function. The physical health summary score (PCS) and mental health summary score (MCS) of the participants were calculated and compared against national standard scores; 50 points were assigned for each summary score. We used the PCS and MCS scores as continuous variables.

##### Depression

The Kessler Psychological Distress Scale (23) is a self-administered screening scale for depression developed by Kessler et al. (24) in the United States. It consists of 10 items and a 5-step Likert scale with a cutoff score of 25 points (23). In this study, participants with a cutoff score ≥25 points were considered depressive, and those with a cutoff score < 25 were considered non-depressive.

##### Family function

The Family and Cohesion Evaluation Scale at Kwansei Gakuin (FACESKG) developed by Tachiki, is a family function evaluation scale based on Olson's Circumplex Model of Marital and Family Systems (25–27). It has two components: family cohesion and family adaptation, with a scoring range of −14.5–19 and range of −13.5–14.5, respectively. Adaptation is interpreted from rigid or structured with lower score to chaotic or flexible with higher score, and cohesion is interpreted as disengaged or separated with low score and enmeshed or connected with higher score (26, 27). We used the scores of adaptation and cohesion as continuous variables.

##### Transition readiness

The Japanese version of the transition Scale (28) was used to assess the participants' transition readiness. It was originally developed by Klassen (29) and consists of three scales: a cancer worry scale (six items, including: “I worry about my cancer every day”), a self-management skills scale (15 items, including: “I know how to contact a doctor if I need to” and “I book my own doctor”s appointment”), and expectations about adult long-term follow-up (LTFU) care scale (12 items, including: “I expect my parent(s) will be able to see the doctor with me” and “I expect the doctor will become like a friend”). The four response options were categorized as “strongly agree (3),” “agree,” “disagree,” or “strongly disagree (0),” and the total score of each scale was calculated. For the cancer worry scale and the self-management skills scale, higher scores indicate high worry or self-management skills, respectively. Regarding the expectations about adult LTFU care scale, the low “Expectation for adult LTFU” score indicates recognition of more independent consultation behavior. The scores of each scale was used as independent variable.

### Procedure

The St. Luke's Lifetime cohort study participants were childhood cancer survivors who received cancer treatment at St. Luke's International Hospital and at hospitals to which the collaborators of this cohort study belonged. Candidates were provided with an explanation of this research from their physicians, and in cases where face-to-face explanations were possible, received verbal explanation of the cohort study. We obtained written consent form when survivors agree to participate in our study. The heath check-up date and questionnaire were mailed to survivors who agreed to participate.

### Analysis

We performed univariate analysis to compare the differences between the regular workers/students and non-regular workers/unemployed groups. The Mann–Whitney *U* test was used for continuous variables (including age and IQ and SF-8, Transition Scale, and FACESKG scores). Chi-square tests were performed on nominal variables (sex, employment status, academic achievement, cancer type, treatment history, physical function, and Kessler Psychological Distress Scale score).

Multivariate logistic regression backward stepwise analysis was performed to determine factors affecting employment status and academic attainment. Variables with *p*­-values <0.1 in the univariate analysis (Mann–Whitney *U* tests or chi-square tests) were used as dependent variables for the multivariate logistic regression analysis. Considering confounding factors, age and sex were included in the model.

All analyses were conducted using Statistical Package for the Social Sciences 28.0 for Macintosh (IBM). A two-sided *p*-value <0.05 was considered statistically significant.

### Ethical consideration

We obtained approval for our research from the Institutional Review Board of St. Luke's International Hospital.

## Results

### Characteristics and descriptive of the survivors

Characteristics of the survivors are shown in Table 1. Of the 68 survivors, 37 (54.4%) were regular workers, 22 (32.4%) were students, and 9 (13.2%) were non-regular workers/unemployed. Annual income significantly differed between the regular workers and non-regular workers (Chi-square test, *p *= 0.005).

**Table 1:** Participants’ characteristics and medical histories.

	Regular worker/Students group				Non-regular worker/Unemployed group	
	Worker (*n* = 37)		Student (*n* = 22)		(*n* = 9)	
**Characteristics (*n* = 68)**						
Age, mean (SD)	29.0	(5.9)	21.5	(3.1)	32.1	(6.5)
Gender (Female), *n* (%)	18	(48.6)	9	(42.9)	5	(55.6)
Academic achievement, *n* (%)						
Graduate school	5	(13.5)	2	(9.1)	1	(11.1)
College	19	(51.4)	16	(72.2)	2	(22.2)
Vocational school/Junior college	5	(13.5)	2	(9.1)	3	(33.3)
High school	6	(16.2)	0	(0.0)	3	(33.3)
Junior high school	2	(5.4)	0	(0.0)	0	(0.0)
**Medical history**						
Cancer type						
Leukemia (*n* = 41)	22	(59.5)	11	(50.0)	8	(88.9)
Neuroblastoma (*n* = 10)	5	(13.5)	4	(18.2)	1	(11.1)
Brain tumor (*n* = 6)	4	(10.8)	2	(9.1)	0	(0.0)
Lymphoma (*n* = 4)	3	(8.1)	1	(4.5)	0	(0.0)
Others (*n* = 7)	3	(8.1)	4	(18.2)	0	(0.0)
Treatment history						
Chemotherapy (*n* = 67)	36	(100.0)	22	(100.0)	9	(100.0)
Surgery (*n* = 22)	13	(36.1)	8	(36.4)	1	(11.1)
Radiation (*n* = 32)	21	(58.3)	5	(22.7)	6	(66.7)
Over Gr3 (*n* = 19)	12	(38.7)	6	(35.3)	1	(25.0)
Hematopoietic cell plantation (*n* = 11)	4	(11.4)	5	(22.7)	2	(22.2)

The most frequently reported physical conditions were dyslipidemia for both the regular workers (14, 35.9%) and non-regular workers/unemployed (5, 55.6%) and followed by dental abnormalities (10, 29.4%; 4, 44.4%, respectively) which is the most frequent for students (8, 38.1%) (Table 2).

**Table 2:** Participant's health conditions and psychosocial statuses.

	Regular workers/Students group				Non-Regular workers/Unemployed group		
	Worker (*n* = 37)		Student (*n* = 22)		(*n* = 9)		
**Health condition (*n* = 68)**							
**Physical condition,** *n* (%)							
Body mass index <18.5	8	(22.1)	7	(31.8)	0	(0.0)	
Hypertension	5	(13.5)	4	(18.2)	1	(11.1)	
Dyslipidemia	14	(35.9)	8	(36.4)	5	(55.6)	
Primary hypothyroidism	2	(5.4)	1	(4.5)	0	(0.0)	
Liver dysfunction	2	(5.4)	2	(9.1)	1	(11.1)	
Kidney dysfunction	1	(2.7)	0	(0.0)	0	(0.0)	
Hepatitis B, C virus infection	4	(10.8)	0	(0.0)	2	(22.2)	
Hearing loss	3	(8.1)	1	(4.5)	2	(22.2)	
Osteoporosis	1	(2.7)	1	(4.5)	1	(11.1)	
Dental abnormalities	10	(29.4)	8	(38.1)	4	(44.4)	
Ocular abnormalities	10	(27.8)	4	(18.2)	1	(11.1)	
Cardiomyopathy	0	(0.0)	1	(4.5)	0	(0.0)	
Gonadal dysfunction	5	(13.9)	2	(9.1)	1	(11.1)	
HPA dysfunctions	6	(17.1)	0	(0.0)	2	(22.2)	
Growth hormone deficiency	5	(13.5)	0	(0.0)	1	(11.1)	
**Neuro-congnitive function, mean (SD)**							
IQ (WAIS-IV)	103.1	(19.1)	107.9	(20.3)	89.0	(14.5)	*
**Psychosocial status, QoL**							
PCS, mean (SD)	26.7	(9.7)	28.5	(15.4)	34.1	(12.5)	*
MCS, mean (SD)	37.5	(11.3)	41.7	(11.3)	44.7	(9.6)	
Depression (K10 = 25), *n* (%)	2	(5.4)	1	(4.5)	1	(11.1)	
**Transition readiness, mean (SD)**							
Transition Scale							
Self-management	32.9	(5.2)	34.6	(5.7)	29.2	(3.7)	*
Expectation of adult LTFU	16.1	(6.1)	15.9	(4.1)	19.3	(2.9)	
Anxiety	5.2	(3.1)	4.3	(3.7)	4.4	(3.3)	*
Knowledge of treatment	11.0	(6.1)	11.0	(6.5)	8.9	(4.9)	
Knowledge of cancer diagnosis	5.5	(2.9)	4.2	(2.9)	5.1	(4.0)	
**Family function (FACESKG), mean (SD)**							
Adaptation	-.87	(2.4)	-.60	(1.3)	−3.1	(2.5)	*
Cohesion	2.0	(3.5)	1.2	(3.1)	1.6	(2.1)	

* *p *< 0.1, Mann-Whitney *U* test between the regular workers/students group and the non-regular workers/unemployed group.

Fifty-three survivors in the regular workers/student group (93%) and nine (81.8%) in the non-regular workers/unemployed group had the SF-8 PCS scores below the national standard score of 50 points. Regarding the MCS, 44 (77.2) and seven (63.3%) participants scored below the national standard score of 50 points (Table 2).

### Univariate analysis

There were no significant differences between the regular workers/student group and the non-regular workers/unemployed group with respect to age, sex, cancer type, treatment histories, or physical function, which were assessed by the comprehensive health check-up (Tables 1, 2). However, IQ was significantly different between the two groups (*p *= 0.012).

In terms of psychosocial indicators, the mean the FACESKG adaptation score was significantly higher in the regular workers/student group than in the non-regular workers/unemployed group (*p *= 0.005), indicating that the family function of the regular workers/student group was more structed than that of the non-regular workers/unemployed group. There was a tendency (*p* < 0.1) of the differences between the SF-8 PCS scores (*p *= 0.073), the Transition Scale “Self-management scale” (*p *= 0.021), and the “Expectation of adult LTFU scale” (*p *= 0.058).

### Multivariate logistic regression

We used IQ results, the SF-8 PCS score, two transition scale scores (“self-management scale” and “expectation of adult LTFU”), and the FACESKG adaptation score, which showed significance or the tendency of difference in the univariate analyses as independent variables for the multivariate logistic regression analysis to determine the predictors; We found no strong correlations between these variables (Table 3). There were four steps till model convergence in multivariate logistic regression. The final model included IQ (odds ratio [OR] = 1.100; 95% confidence interval [CI] 1.015–1.193; *p *= 0.021) (Table 4), the FACESKG adaptation score (OR=2.337; 95% CI 1.175–4.645; *p *= 0.015), the “Expectation of adult LTFU” score (OR = 0.612; 95% CI 0.396–0.974; *p *= 0.038), and the “Self-Management” score (OR = 1.279; 95% CI 0.960–1.193; *p *= 0.093).

**Table 3:** Correlations between independent variables.

	*n* = 68			
	IQ	TS self-management	TS expectation	FACES adaptation
TS Self-management	0.115			
TS Expectation	0.021	−0.212		
FACESKG Adaptation	0.029	−0.073	0.011	
QOL PCS	0.103	−0.104	0.068	0.002

Spearmans's Rho.

TS, the transition Scale.

**Table 4:** Multivariable logistic regression for employment.

						*n* = 67
	**Step 1**			**Step 2**		
	OR	95% Cl	*p*-value	OR	95% Cl	*p*-value
IQ	**1**.**096**	**1.010**–**1.190**	**0**.**028**	**1**.**100**	**1.015**–**1.193**	**0**.**021**
SF-8 PCS	0.951	0.873–1.037	0.225			
TS						
Self-MGT	1.281	0.955–1.171	0.098	1.279	0.960–1.193	0.093
Expectation	0.637	0.401–1.011	0.056	**0**.**612**	**0.396**–**0.974**	**0**.**038**
FACESKG						
Adaptation	**2**.**419**	**1.159**–**5.047**	**0**.**019**	**2**.**337**	**1.175**–**4.645**	**0**.**015**

Multivariate logistic regression was performed using the stepwise likelihood method.

OR, odds ratio; TS, the transition scale; Self-MGT, self-management.

## Discussion

This study aimed to explore factors associated with childhood cancer survivors' employment status and academic achievement. Although the number of subjects was small, valuable data were obtained, and we found that IQ, transition readiness, and family function associated with employment status and academic attainment.

The St. Jude survey (2) reported that more than 98% of childhood cancer survivors have conditions in their 30 s, while on the contrary, this study observed that around 85% of survivors have these conditions. These differences in frequency may be because the participants in this study were younger than those in the St. Jude survey, and the frequency of associated conditions may increase in this cohort in the future. This study's most frequently occurring late effects were dental abnormalities, low weight, ophthalmic abnormalities, and dyslipidemia, which concurred with previous studies (2). Dental and ophthalmic abnormalities are not usually examined in long-term follow-ups centered on pediatrics and hematology. However, this study revealed that these were common late effects, so regular examinations of these are required with the establishment of an insurance-covered follow-up strategy. Additionally, it is clear that childhood cancer survivors in Japan have late effects similar to that observed in survivors belonging to other countries, and their framework of care is considered more seriously than ever before.

According to the Labour Force Survey 2020 by the Statistics Bureau of Japan (30), the percentage of regular workers aged 25–34 years is 77.6%, while the percentage of regular workers in this study is slightly higher at 81.8%. Similarly, regarding academic attainment, 62.3% of this study's subjects graduated from or were enrolled in university or graduate school, and the achievement is higher than Japanese population rate of 41.9%. However, this result needs to be interpreted carefully. This study was conducted in the metropolitan area of Tokyo, in which many of its subjects lived. The Tokyo metropolitan area is reported to have a higher university enrollment rate than other prefectures, which is 64.7% (31). Therefore, this study's high rate of university enrollment may be related to the region's characteristics. Previous population survey reported lower rate of the survivors' university enrollment than that of the general population. Therefore, it can be said that the employment form and academic achievement of childhood cancer survivors are equivalent to that of the metropolitan area's general population.

In terms of factors related to employment status, we did not find any significant associations with diagnosis, treatment, or physical condition, screened using the CTCAE. The most frequent late effects in our study were dyslipidemia and dental abnormalities. These late effects need condition management or regular clinic visits to prevent their progression; however, these may not substantially impact work or school performance. Therefore, further study is needed to clarify the association of what late effects on employment or academic attainment.

A previous study showed that more adaptive family functioning was associated with better outcomes in brain tumor survivors (32). Our study family function is the strongest variable and showed that more structured family adaptation was associated with better states of survivor employment and academic attainment, so patients' families should handle their affected loved ones' situations consistent way and make decisions according to the survivor's developmental stage. It is often reported that childhood cancer survivors experience parent-child closeness and overprotection. Therefore, family's, especially parental adaptation influences on CCSs's adaptation. It is important to encourage the gradual transfer of clinical decision-making from parents to survivors and help survivors make decisions by providing the necessary information and coping methods with consistent way according to the patients' cognitive functions, such as IQ and developmental stages. Besides support from family and health care providers, interaction with peers or support in the school environment could be significant (33–35); child onset cancer survivors diagnosed before forming peer-group friendships overcome their cancer treatment with their parents' support. Therefore, these patients developed a strong bond and feeling of trust with their parents. In adolescent and young adult survivors, on the other hand, it is reported that talking with peers is an important form of social support (36); therefore, it is possible that interacting with peers allows survivors to gain familiar role models and goals from their peers, and encouraging appropriate interaction with peers may positively affect future employment.

As previous studies, IQ showed the strong association with employment status in this study. It is widely known that treatment for childhood cancer impairs cognitive function. It has been reported that cognitive impairment affects study, social interaction, employment, and self-management of one's body (37), and this study found that it also affects employment and academic performance achievement. In the multivariate logistic analysis, self-management, readiness of independent medical use (measured by expected for adult medical care), and structured family function contributed to employment and academic achievement. Strengthening consistent family function may have a positive impact on the independence of survivors against treatment-induced decline in IQ. However, this study's sample size was small and did not examine the positive effects of family function on IQ. Further research is needed to explore the positive impact on family function considering various contexts such as IQ levels. In the regular workers/students group, “Expectation for adult LTFU” scores were lower than those in the non-regular workers/unemployed group, meaning that survivors with regular workers/students have different expectations regarding adult medical clinic visits. Thus, the low “Expectation for adult LTFU” score indicates recognition of more independent consultation behavior; in other words, the participants' preparation for the transition from pediatric care to adult care was high. It is thought that such independence and decision-making ability in medical use will function positively for making choices to suit themselves and will have the same positive effect on academic attainment and employment.

Similar to previous research (38), the participants' average QoL scores were below the national standard scores for both PCS and MCS. This shows that childhood cancer survivors experience subjective difficulties with their physical and mental health. Interestingly, we found no differences in QoL scores between the regular workers/students and non-regular workers/unemployed groups, but there was a tendency for lower PCS scores in survivors in the regular workers/students group. Full-time work may impose a physical burden, therefore, further research regarding employment and subjective physical burden will provide additional details and recommendations for continuation of employment measures.

There are some limitations to this study. The first is its small sample size, the primary reason for which was the high cost of providing comprehensive health check-ups. Fortunately, our facility has a preventive health center specializing in these, and we obtained support from multidisciplinary health care providers and several foundations to carry out comprehensive health checks not covered by health insurance. Also, although as strength of this study was its method, which included comprehensive health check-ups, there were time and financial barriers to participation for survivors if they lived far from our hospital. For this reason, we began surveying childhood cancer survivors treated at our institution and gradually expanded the recruitment to subjects treated at other hospitals. However, due to the small number of samples, many subjects were regular workers/students, and the statistical power of our results is limited. In that sense, the results of this study will be strengthened by repeated verifications in the future.

Another limitation is our method of grouping employment type. In this study, we combined childhood cancer survivors in regular workers with those who were students (20 were college or graduate students, and 2 were junior college or vocational college) into one group. Strictly speaking, students are promised to be a regular workers; however, in Japan, about 80% of university graduates and 90% of graduate school graduates work as a regular worker (30). Based on this, we treated these as one group. In addition, our results also showed a relationship between university or graduate school graduation and full-time employment (*p *= 0.049).

This study aimed to gain insight into the perceptions of childhood cancer survivors to obtain suggestions for biopsychosocial survivorship care. Considering its limitations, we think of this as a preliminary study, and we will increase the number of subjects to report the employment status of Japanese childhood cancer survivors with a sufficient number of participants in the future. As mentioned above, we will recruit survivors treated at our hospital and those treated at collaborating institutions.

We examined the employment of childhood cancer survivors and clarified the association of biological and psychosocial functions with employment. Due to the high frequency of physical symptoms revealed by the rigorous health check-ups in this study, comprehensive examinations are required for long-term follow-up and provide complete care for survivors' overall independence. This study clarified the importance of supporting their families and improving patients' readiness for transition to self-management. In Europe, the PanCareFollowUp consortium has been established and is implementing follow-up strategies, including patient-centric care-based lifestyle interventions and coaching (39). The St. Luke's lifetime cohort study also provides feedback of results of health check-up and the necessary guides for long-term follow-up in consideration of individual biopsychosocial state. We believe that it is important to further promote such research and clinical practice in an integrated manner to support better survivorship of survivors.

## Conclusion

We examined childhood cancer survivors’ employment and academic status and found that family function showed strongest association followed by IQ with employment status. Furthermore, transition readiness was associated with survivors' employment and academic status. We, therefore, recommend that long-term follow-up provides total care with support for childhood cancer survivors' physical, psychological, and social functions to improve health, readiness for transition to self-management, and family functioning.

## Data Availability

The raw data supporting the conclusions of this article will be made available by the authors, without undue reservation.

## References

[1] Schwartz C Hobbie W Constine L Ruccione K. Survivors of childhood and adolescent cancer. Berlin: Springer (2005).

[2] Bhakta N Liu Q Ness KK Baassiri M Eissa H Frederick Y The cumulative burden of surviving childhood cancer: an initial report from the st. Jude lifetime cohort study. Lancet. (2017) 390:2569–82. 10.1016/S0140-6736(17)31610-0 28890157 PMC5798235

[3] Lancashire ER Frobisher C Reulen RC Winter DL Glaser A Hawkins MM. Educational attainment among adult survivors of childhood cancer in Great Britain: a population-based cohort study. J Natl Cancer Inst. (2010) 102:254–70. 10.1093/jnci/djp498 20107164

[4] Peterson CC Johnson CE Ramirez LY Huestis S Pai ALH Demaree HA A meta-analysis of the neuropsychological sequelae of chemotherapy-only treatment for pediatric acute lymphoblastic leukemia. Pediatr Blood Cancer. (2008) 51:99–104. 10.1002/pbc.21544 18322925

[5] Campbell LK Scaduto M Sharp W Dufton L Slyke DV Whitlock JA A meta-analysis of the neurocognitive sequelae of treatment for childhood acute lymphocytic leukemia. Pediatr Blood Cancer. (2007) 49:65–73. 10.1002/pbc.20860 16628558

[6] Boman KK Lindblad F Hjern A. Long-term outcomes of childhood cancer survivors in Sweden: a population-based study of education, employment, and income. Cancer. (2010) 116:1385–91. 10.1002/cncr.24840 20087961

[7] Soejima T Sato I Takita J Koh K Kaneko T Inada H Impacts of physical late effects on presenteeism in childhood cancer survivors. Pediatr Int. (2020) 62:1241–9. 10.1111/ped.14293 32402092

[8] Jacola LM Baran J Noll RB Willard VW Hardy KK Embry L Adaptive functioning and academic achievement in survivors of childhood acute lymphoblastic leukemia: a report from the Children's Oncology group. Pediatr Blood Cancer. (2021) 68:e28913. 10.1002/pbc.28913 33522102 PMC8212574

[9] Harshman LA Barron S Button AM Smith BJ Link BK Lynch CF Population-based exploration of academic achievement outcomes in pediatric acute lymphoblastic leukemia survivors. J Pediatr Psychol. (2012) 37:458–66. 10.1093/jpepsy/jsr119 22271791 PMC3334536

[10] Phipps S Rai SN Leung W Lensing S Dunavant M. Cognitive and academic consequences of stem-cell transplantation in children. J Clin Oncol. (2008) 26:2027–33. 10.1200/JCO.2007.13.6135 18421056

[11] Teckle P Peacock S McBride ML Bentley C Goddard K Rogers P. Long-term effects of cancer on earnings of childhood, adolescent and young adult cancer survivors – a population-based study from British Columbia, Canada. BMC Health Serv Res. (2018) 18:826. 10.1186/s12913-018-3617-5 30382843 PMC6211561

[12] Frobisher C Lancashire ER Jenkinson H Winter DL Kelly J Reulen PC Employment status and occupational level of adult survivors of childhood cancer in Great Britain: the British childhood cancer survivor study. Int J Cancer. (2017) 140:2678–92. 10.1002/ijc.30696 28316069 PMC5434894

[13] Berbis J Reggio C Michel G Chastagner P Bertrand Y Kanold J Employment in French young adult survivors of childhood leukemia: an LEA study (for leucemies de l’Enfant et de l’Adolescent—childhood and adolescent leukemia). J Cancer Surviv. (2016) 10:1058–66. 10.1007/s11764-016-0549-0 27185246

[14] Frederiksen E Pedersen C Mogensen H Mader L Bautz A Talback M Employment status and occupational positions of childhood cancer survivors from Denmark, Finland and Sweden: a nordic register-based cohort study from the SALiCCS research programme. Lancet Reg Health Eur. (2022) 12:100258. 10.1016/j.lanepe.2021.100258 34901911 PMC8640515

[15] Haupt R Spinetta JJ Ban I Barr RD Beck JD Byrne J Long term survivors of childhood cancer: cure and are. The erice statement. Eur J Cancer. (2007) 43:1778–80. 10.1016/j.ejca.2007.04.015 17543517

[16] Jankovic M Haupt R Spinetta JJ Beck JD Byrne J Calaminus G Long-term survivors of childhood cancer: cure and care–the erice statement (2006) revised after 10 years (2016). J of Cancer Surviv. (2018) 12:647–50. 10.1007/s11764-018-0701-0 29946794

[17] Survey on Employment of Young People. Employment, wage and labour welfare statistics office. Tokyo: Ministry of Health, Labour and Welfare (2013). https://www.mhlw.go.jp/toukei/list/4-21c-jyakunenkoyou-h25.html [Accessed May 22, 2021].

[18] The Census. The ministry of internal affairs and communications. Tokyo: Health, Labour and Welfare Statistics Association (2010). https://www.stat.go.jp/data/kokusei/2010/pdf/chouhyou.pdf [Accessed May 22, 2021].

[19] Japan Clinical Oncology Group. Common terminology criteria for adverse events (CTCAE) version 4.0. Tokyo: Japan Clinical Oncology Group (2010). http://www.jcog.jp/doctor/tool/CTCAEv4J_20100911.pdf [Accessed September 21, 2022].

[20] U.S. Department of Health and Human Services, National Institutes of Health, National Cancer Institute. Common terminology criteria for adverse events (CTCAE) version 4.0. Maryland: U.S. Department of Health and Human Services (2009). https://evs.nci.nih.gov/ftp1/CTCAE/CTCAE_4.03/CTCAE_4.03_2010-06-14_QuickReference_5 × 7.pdf [Accessed September 21, 2022].

[21] Yoshimoto-Suzuk Y Hasegawa D Hosoya Y Saito G Nagase K Gunji M Significance of active screening for detection of health problems in childhood cancer survivors. Front Pediatr. (2022) 10:947646. 10.3389/fped.2022.947646 36275067 PMC9583383

[22] Fukuhara S Suzukamo Y. Instruments for measuring health-related quality of life -SF8 and SF36. J Ciln Experi Med. (2005) 213:133–6. (In Japanese). https://www.ishiyaku.co.jp/magazines/ayumi/AyumiBookDetail.aspx?BC=921302

[23] Furukawa T Ono Y Uda H Nakane Y. A study on simple screening of mental illness in the general population. In: Kawakami K, editors. Report on the research on mental health problems and the actual situation of countermeasure infrastructure. Tokyo: Report of the Ministry of Health Labour and Welfare, Special research (2003) 127–30. (In Japanese).

[24] Kessler RC Andrews G Colpe LJ Hiripi E Mroczek DK Normand SLT Short screening scales to monitor population prevalences and trends in nonspecific psychological distress. Psychol Med. (2002) 32:959–76. 10.1017/S0033291702006074 12214795

[25] Olson DH. FACES IV and the circumplex model. J Marital Fam Ther. (2011) 3:64–80. 10.1111/j.1752-0606.2009.00175.x 21198689

[26] Olson DH Russell CS Sprenkle DH. Circumplex model: Systemic assessment and treatment of families. New York: Haworth Press (1989).

[27] Tachigi S. Theoretical and empirical verification of the family system-examination of the validity of Olson's Circumplex model. Tokyo: Kawashima Shoten (1999). (In Japanese).

[28] Ishida Y Tezuka M Hayashi M Inoue F. Japanese Childhood cancer survivors’ readiness for care as adults: a cross-sectional survey using the transition scales. Psychooncology. (2017) 26:1019–26. 10.1002/pon.4276 27598031

[29] Klassen AF Rosenberg-Yunger ZRS D’Agostino NM Cano SJ Barr R Syed I The development of scales to measure childhood cancer survivors’ readiness for transition to long-term follow-up care as adults. Health Expect. (2015) 18:1941–55. 10.1111/hex.12241 25052198 PMC5810698

[30] Labor Force Survey. Statistics bureau of Japan. Tokyo: Health, Labour and Welfare Statistics Association (2010). https://www.stat.go.jp/data/roudou/sokuhou/nen/ft/pdf/index1.pdf [Accessed May 22, 2021].

[31] Report on School Basic Survey. Ministry of education, culture, sports, science and technology. Tokyo: Ministry of Education, Culture, Sports, Science and Technology (2020). https://www.e-stat.go.jp/stat-search/files?page=1&layout=datalist&toukei=00400001&tstat=000001011528&cycle=0&tclass1=000001148386&tclass2=000001148404&tclass3=000001148419&tclass4=000001148423&tclass5= 000001148424 [Accessed May 22, 2021].

[32] Moscato E Patronick J Wade SL. Family functioning and adaptation following pediatric brain tumor: a systematic review. Pediatr Blood Cancer. (2022) 69:e29470. 10.1002/pbc.29470 34842339

[33] Stegenga K Ward-Smith P. The adolescent perspective on participation in treatment decision making: a pilot study. J Pediatr Oncol Nurs. (2008) 25:112–7. 10.1177/1043454208314515 18270308

[34] Smith AW Parsons HM Kent EE Bellizzi K Zebrack BJ Keel G AYA HOPE study collaborative group. Unmet support service needs and health-related quality of life among adolescents and young adults with cancer: the AYA HOPE study. Front Oncol. (2013) 8:75. 10.3389/fonc.2013.00075 PMC3619248 23580328

[35] Zebrack BJ Block R Hayes-Lattin B Embry L Aguilar C Meeske KA Psychosocial service use and unmet need among recently diagnosed adolescent and young adult cancer patients. Cancer. (2013) 119:201–14. 10.1002/cncr.27713 22744865

[36] Kent E Wilder-Smith A Keegan TH Lynch CF Wu X Hamilton AS Talking about cancer and meeting peer survivors: social information needs of adolescents and young adults diagnosed with cancer. J Adolesc Young Adult Oncol. (2013) 2:44–52. 10.1089/jayao.2012.0029 23781400 PMC3684139

[37] Vetsch J Wakefield CE McGill BC Cohn RJ Ellis SJ Stefanic N Educational and vocational goal disruption in adolescent and young adult cancer survivors. Psychooncol. (2018) 27:532–8. 10.1002/pon.4525 28778113

[38] Schulte FSM Chalifour K Eaton G Garland SN. Quality of life among survivors of adolescent and young AdultCancer in Canada: a young adults with cancer in their prime (YACPRIME) study. Cancer. (2021) 127:1325–33. 10.1002/cncr.33372 33332603

[39] Kalsbeeka RJ van der Pal HJH Hjorth L Wintherd JF Michel G Haupt R The European multistakeholder PanCareFollowUpproject: novel, person-centred survivorship care to improve care quality, effectiveness, cost-effectiveness and accessibility for cancer survivors and caregivers. Eur J Cancer. (2021) 153:316–28. 10.1016/j.ejca.2021.06.004 34153717

